# Supplementary material for: Lung Transplantation in Idiopathic Pulmonary Fibrosis Patients in the European MultiPartner IPF Registry: Challenges for Health Equity
Source: Biomedicines. 2025 Oct 31;13(11):2684. doi: 10.3390/biomedicines13112684 (PMC12650749; doi:10.3390/biomedicines13112684)
Supplement: Supplementary file 1 [file biomedicines-13-02684-s001.zip › biomedicines-3912418-supplementary.pdf]

Supplementary Material

Questionnaire S1.

Lung transplantation country specific questions for EMPIRE Steering Committee members

1. Does your country have a LuTX program? (yes/no)

2. Does your center evaluate IPF patients for LuTX? (yes/no)

3. Does your program use ISHLT criteria for TX, or more strict criteria are used?

4. What is the suggested age limit for IPF patients for LuTX?

5. Please rank the following as being the most important to the least important factors for no referral of ***IPF patients*** to LuTX

**Condition**	**Rank (1 most important, 14 least important)**
Age >65 years	
Age >70 years	
Current smoking	
Social deprivation	
Comorbidity of heart failure	
Comorbidity of renal failure	
Comorbidity of osteoporosis	
Comorbidity of liver cirrhosis	
Cancer any type	
Positron emisison tomographic positive changes requiring additional invasive investigations	
Positive panel-reactive antibodies	
Autoimmune serology positivity	
Body mass index >35 kg/m^2	
Body mass index <16 kg/m^2	

6. What was the main reason for not listing IPF patients you referred for LuTX?

Please list maximum of 5 reasons:

Table S1. Centers of the EMPIRE registry and the number of transplanted patients by center

Country	Center	Number of LuTx (N=94)	Questionnaire returned (yes/no)
Austria	Clinical Research Center Salzburg GmbH	2 (2.1%)	yes
	Kepler University Hospital, Department of Pulmonology, Linz	0	
Bulgaria	Acibadem City Clinic Tokuda Hospital, Pulmonary Department, Sofia	0	no
Croatia	Split University Hospital Center, Department of Pulmonology, Split	0	no
	University Hospital Dubrava, Department of Pulmonology, Zagreb	1 (1.1%)	
Czech Republic	Thomayer University Hospital, Dept. of Pulmonary Medicine	6 (6.4%)	yes
	Hospital Na Bulovce, Dept. of Pulmonary Medicine and Thoracic Surgery	0	
	University Hospital in Motol, Dept. of Pulmonary Medicine	1 (1.1%)	
	University Hospital Plzen, Dept. of Pneumology and Phthisiology	2 (2.1%)	
	University Hospital Hradec Kralove, Dept. of Pulmonary Medicine	2 (2.1%)	
	Regional Hospital Pardubice, Dept. of Pulmonary Diseases and TB	0	
	University Hospital Olomouc, Dept. of Pulmonary Diseases and TB	4 (4.3%)	
	Hospital Kromeriz, Dept. of Pulmonary Medicine and Allergology	0	
	University Hospital Brno, Dept. of Pulmonary Diseases and TB	0	
	Hospital Znojmo, Dept. of Pulmonary Medicine	0	
	Hospital Jihlava, Dept. of Pulmonary Medicine	0	
	Masaryk Hospital in Usti nad Labem, Dept. of Pulmonary Medicine	0	
	Hospital Ceske Budejovice, Dept. of Pulmonary Medicine and TB	0	
	University Hospital Ostrava, Dept. of Pulmonary Diseases and TB	5 (5.3%)	
	Hospital Novy Jicin, Dept. of Pneumology and Phthisiology	0	
	Tomas Bata Regional Hospital, Dept. of Pulmonary Medicine	0	
Hungary	National Korányi Tuberculosis and Pulmonology Institute, Department of Pulmonology, Budapest	0	yes
	Semmelweis University, Department of Pulmonology, Budapest	8 (8.5%)	
	Debrecen University Clinical Centre, Centre for Pulmonology, Debrecen	0	

	Petz Aladár County Teaching Hospital, Department of Pulmonology, Győr	0	
	Pécs University, Department of Pulmonology, Pécs	0	
	University of Szeged, Department of Pulmonology, Szeged	0	
Israel	Clalit Health Services through the Rabin Medical Centre, Institute of Pulmonary Medicine, Petah Tikva	53 (56%)	yes
	Carmel Medical Center, Haifa	0	
North Macedonia	Clinic of Pulmonology and Allergology, Skopje	0	no
Poland	University Hospital Gdańsk, Allergology and Pneumonology Department, Gdańsk	4 (4.3%)	yes
	Medical University of Silesia, School of Medicine in Katowice, Katowice	0	
	Jagiellonian University, School of Medicine, Department of Pulmonology, Kraków	0	
	Medical University of Łódź, Department of Pneumonology and Allergology, Łódź	0	
	Poznan University of Medical Sciences, Poznań	0	
	Institute of Tuberculosis and Lung Diseases, 1st Department of Pulmonary Diseases, Warsaw	2 (2.1%)	
	Institute of Tuberculosis and Lung Diseases, 2nd Department of Pulmonary Diseases, Warsaw	0	
	Institute of Tuberculosis and Lung Diseases, 3rd Department of Pulmonary Diseases, Warsaw	0	
	Medical University of Warsaw, Department of Internal Medicine, Pulmonary Diseases and Allergy, Warsaw	0	
Serbia	School of Medicine Belgrade, Clinic for Pulmonology,	0	yes
	Clinical Centre of Serbia, Belgrade	0	
	Clinic for Lung Diseases Knes Selo, Clinical Centre of Nis, Niš	0	
	Institute for Pulmonary Diseases of Vojvodina,	0	
	Clinic for Tuberculosis and Granulomatous Diseases, Sremska Kamenica	0	
	Clinical Centre Kragujevac, Clinic for Pulmonology, Kragujevac	0	
Slovakia	University Hospital Bratislava	0	no
	L. Pasteur University Hospital Košice	0	
	ZAPA JJ Ltd., Pneumology and Phthisiology Outpatient Centre, Levice	0	
	University Hospital Martin	0	
	St. Svorad's Specialised Hospital Zobor, Nitra	0	

	National Institute of Tuberculosis, Lung Disorders and Thoracic Surgery Vyšné Hagy	2 (2.1%)	
	F. D. Roosevelt University Hospital in Banská Bystrica	0	
Turkey	Ege University Medical School, Department of Pulmonary Medicine, Izmir	2 (2.1%)	no
